# Highly Regular LIPSS on Thin Molybdenum Films: Optimization and Generic Criteria

**DOI:** 10.3390/ma16072883

**Published:** 2023-04-04

**Authors:** Juraj Sládek, Kryštof Hlinomaz, Inam Mirza, Yoann Levy, Thibault J.-Y. Derrien, Martin Cimrman, Siva S. Nagisetty, Jan Čermák, The Ha Stuchlíková, Jiří Stuchlík, Nadezhda M. Bulgakova

**Affiliations:** 1 HiLASE Centre, Institute of Physics of the Czech Academy of Sciences, Za Radnicí 828, 252 41 Dolní Břežany, Czech Republic; 2 Faculty of Nuclear Sciences and Physical Engineering, Czech Technical University in Prague, Trojanova 13, 120 00 Prague, Czech Republic; 3 Coherent Laser Systems GmbH & Co. KG, Hans Boeckler Str. 12, 37079 Göttingen, Germany; 4 Institute of Physics of the Czech Academy of Sciences, Cukrovarnická 10, 162 00 Prague, Czech Republic

**Keywords:** laser-induced periodic surface structures, highly regular LIPSS, thin film, molybdenum

## Abstract

A systematic experimental study was performed to determine laser irradiation conditions for the large-area fabrication of highly regular laser-induced periodic surface structures (HR-LIPSS) on a 220 nm thick Mo film deposited on fused silica. The LIPSS were fabricated by scanning a linearly polarized, spatially Gaussian laser beam at 1030 nm wavelength and 1.4 ps pulse duration over the sample surface at 1 kHz repetition rate. Scanning electron microscope images of the produced structures were analyzed using the criterion of the dispersion of the LIPSS orientation angle (DLOA). Favorable conditions, in terms of laser fluence and beam scanning overlaps, were identified for achieving DLOA values <10${}^{\circ }$. To gain insight into the material behavior under these irradiation conditions, a theoretical analysis of the film heating was performed, and surface plasmon polariton excitation is discussed. A possible effect of the film dewetting from the dielectric substrate is deliberated.

## 1. Introduction

In recent years, the fabrication of highly regular laser-induced periodic surface structures (HR-LIPSSs) over large areas using ultrashort laser pulses has been a topic of interest due to their scientific and industrial applications [1,2,3]. Self-replication of the LIPSS makes it possible to cover material surfaces with ripples by scanning the laser beam with appropriate parameters [1,4,5,6,7,8,9,10]. More recently, emphasis was set on uniformity [7,11,12] and on the regularity of the LIPSS over the irradiated sample surface that can be particularly important for the fabrication of phase elements, sensors, cell growth, and surface coloration. For example, Öktem et al. [13] achieved highly regular structures by scanning the laser over thin Ti films, to create oxidation-driven LIPSS. Ruiz de la Cruz et al. [14] obtained relatively uniform large-area LIPSS, fabricated on a 1 µm thick Cr film deposited on a composite substrate. Gnilitskyi et al. [15] reported HR-LIPSS fabricated on large surface areas of various metals, some of which are in the form of several hundreds of nm thick films deposited on quartz glass. Recently, very high regularity could be obtained using direct laser interference processing on a stainless-steel surface [16], while the spatial period with this technique is restrained by the diffraction limit. Other groups achieved high regularity on thin metallic films, revealing their potential when the thickness is about or smaller than the optical penetration depth [17,18]. In Ref. [15], the physical origin of regularity was linked to a decay length of excited surface plasmon polaritons (SPP), and the dispersion of the LIPSS orientation angle (DLOA) proposed in that study is now often used as a regularity criterion [19,20,21,22,23].

In the present study, we performed a detailed investigation of LIPSS formation on 220-nm thick Mo films deposited on amorphous SiO${}_{2}$ substrates. To find the trends toward optimal laser irradiation conditions for HR-LIPSS formation, several raster scans were performed by varying the laser fluence and overlap between lasers spots. Within the range of experimentally determined irradiation conditions for HR-LIPSS fabrication, their regularity was further investigated by changing the laser polarization orientation with respect to the scanning direction. A theoretical analysis of the Mo film laser heating and SPP decay length is performed to better understand material behavior under our irradiation conditions.

## 2. Materials and Methods

### 2.1. Experimental Setup

Mo thin film samples were irradiated using the PERLA–B laser (λ=1030 nm wavelength, τ=1.4 ps FWHM pulse duration, *f* = 1 kHz repetition rate). PERLA–B is one of the diode-pumped solid state (Yb:YAG) pulsed laser systems developed at the HiLASE Center [24]. It is a relatively compact laser, which was already successfully used in various applications [25,26]. In the series of experiments reported here, the output pulse energy was controlled using a combination of a λ/2 wave plate and a pair of thin film polarizers.

For large-area laser scanning, the beam was delivered on the sample surface through a galvo scanner (Scanlab, Intelliscan 20) with an f-theta lens (focal length 163 mm). The 1/$e^{2}$ Gaussian beam diameter 2$w_{0}$, on the sample surface [27] was 32.5 µm. To find suitable scanning parameters for regular and homogeneous LIPSS formation on the sample, areas of 0.3×0.15 mm${}^{2}$ were scanned at various irradiation conditions that include varying the peak fluence $F_{p}$, overlaps between successive laser pulses (*x* direction) and between scanning lines (*y* direction), polarization orientation, and scanning pattern. The overlaps along the *x* and *y* directions were calculated using the expressions $O_{x}=\left(1-V_{x}/\left(2w_{0}f\right)\right)$ and $O_{y}=\left(1-\Delta y/\left(2w_{0}\right)\right)$, respectively, where $V_{x}$ is the scanning speed and Δy is the distance between the centers of scanning lines. The effective number *N* of laser pulses per irradiation spot can be estimated from the expression involving both overlaps $N=1/[\left(1-O_{x}\right)\left(1-O_{y}\right)]$.

In view of the large number of influencing parameters, we restricted the studies to changing only one parameter for each set of scanning. The polarization orientation was thus investigated only for a fixed regime of overlap and fluence. The effect of both overlaps was studied at a fixed fluence of 0.266 J/cm${}^{2}$. This choice is conditioned by our preliminary studies, which revealed that, at certain overlaps, this fluence is near optimal to yield HR-LIPSS. Finally, the influence of the scanning pattern (uni- or bi-directional scanning) on the LIPSS regularity was tested at fixed overlaps $O_{x}$ and $O_{y}$, close to but not exactly at the ideal conditions. Table 1 summarizes the different tests performed in this study.

### 2.2. Materials

Mo thin films were prepared by magnetron sputtering from a Mo target of 60-mm diameter on fused silica substrates. After pumping and degassing the deposition chamber down to a pressure of 10${}^{-4}$ Pa, argon (purity 6.0, flow rate 2 sccm) was used for sputtering at a pressure of 1.1 Pa. When the sputtering conditions were stabilized after the first 5 min, the screen above the target was removed, and molybdenum was deposited on the substrates. The thickness of the sample of 220 nm was measured by an alpha step. The deposition rate of 0.18 nm/sec was evaluated from the film thickness and the deposition time of 20 min. In our custom-made magnetron, we used a DC voltage of 425 V at a current of 0.1 A. The distance between the target and substrates was 80 mm.

**Figure 1:**
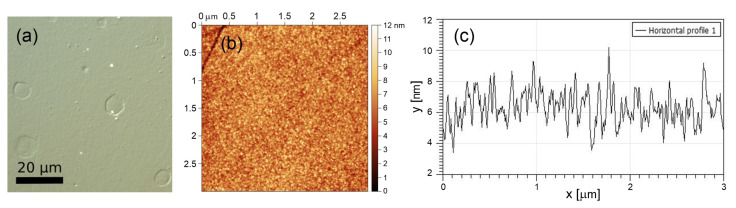
Differential interference contrast optical microscope image (**a**) and a 3 × 3 µm AFM image (**b**) of the non-irradiated surface of the Mo film. (**c**) Surface roughness profile in the middle of the AFM image shown in (**b**).

Figure 1 shows the surface state of non-irradiated parts of the 220 nm thick Mo film. On the differential interference contrast microscope image (left), shallow round defects are seen. Additionally, micrometer-size particles are present on the film surface. It is known that, even for ultra-high vacuum physical vapor deposition conditions, some defects can be formed due to imperfection of the substrate surface or its contamination by foreign particles before the film growth or during deposition [28,29]. Below we show how such kind of defects can affect the LIPSS regularity. The atomic force microscope (AFM) image (Figure 1 right) taken before laser irradiation shows that the root mean square roughness of the film surface was ∼1 nm (as measured by an analysis of a 3 × 3 µm${}^{2}$ area).

### 2.3. Regularity Analysis Using the Dispersion of the LIPSS Orientation Angle (DLOA)

To characterize the LIPSS regularity, we analyzed the dispersion of the LIPSS orientation angle [15] for selected scanning electron microscope (SEM) images. The DLOA is a repeatable and practical technique to evaluate LIPSS regularity, which is more precise as compared to the evaluation of an approximate angular opening of the two-dimensional fast Fourier transform (2D-FFT). To compare the DLOA for different irradiation conditions, all SEM (1024 × 1024 pixels) images were acquired with the same acquisition settings. Low acquisition speed was used to reduce image noise. The field of view, 50 × 50 µ$m^{2}$, was chosen large enough to allow sufficient statistics of the DLOA.

## 3. Results

### 3.1. Effect of Polarization Orientation

**Figure 2:**
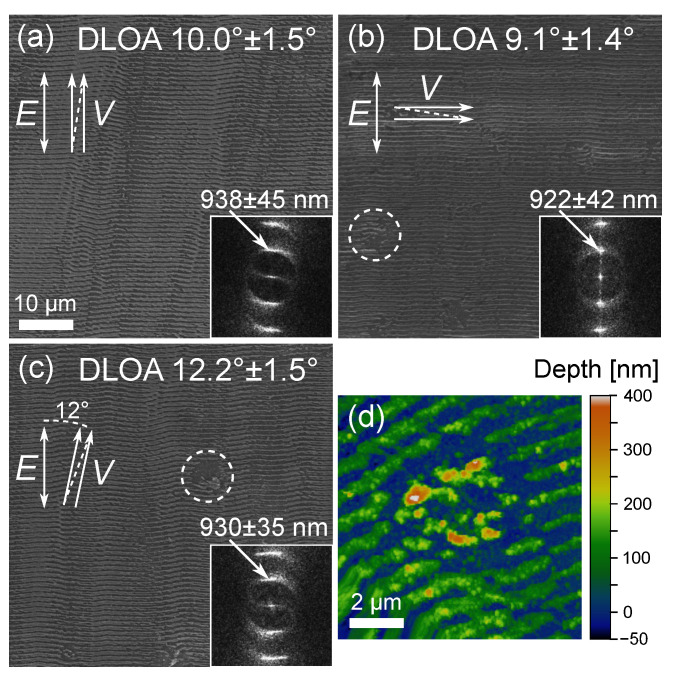
(**a**–**c**) Morphology and DLOA of the LIPSS obtained at three different orientations of laser polarization (*E*) relative to laser scanning direction (*V*). The inset in each SEM image shows corresponding 2D-FFT with LIPSS periodicity indicated. In all cases, laser irradiation parameters were: $F_{p}$ = 0.266 J/cm${}^{2}$, $O_{x}$ = 92.3 %, Δy = 8 µm, $O_{y}$ = 75.4 %, effective number of pulses per irradiation spot N∼53. (**d**) AFM image of one of the strongly ablated areas. Such areas are highlighted by dotted circles in (**b**,**c**).

Figure 2 shows the morphology of LIPSS prepared on the Mo thin films for three different orientations of polarization with respect to the beam scanning direction (Figure 2a–c). In the insets, the corresponding 2D-FFT images are shown with indications of the DLOA and polarization direction. In all cases, the produced LIPSS were oriented perpendicular to the incident beam polarization as generally observed for metals. At 0.266 J/cm${}^{2}$ and with $O_{x}$ and $O_{y}$ close to the optimal region of overlaps (see Section 3.2), the minimum DLOA, 9.1${}^{\circ }$, is obtained when the laser scanning direction is perpendicular to the laser light polarization direction. The case with the polarization parallel to the scanning direction exhibits also an acceptable DLOA of 10${}^{\circ }$. When changing the angle of the polarization with respect to the scanning direction (Figure 2c), the DLOA was typically increasing under our irradiation conditions. The spatial periods of the LIPSS in these three scans are very similar, 938, 922, and 930 nm, respectively, for the scanning directions parallel, perpendicular to polarization, and at the angle of 12${}^{\circ }$ to it.

In Figure 2b,c, strongly ablated areas (∼5 µm in diameter, outlined by dashed circles) with discontinuous LIPSS can be seen. A detailed AFM scan of such an area is shown in Figure 2d. As mentioned above, defects of a similar size were present on the surface prior to irradiation. During the raster scanning, the enhanced absorption of laser light on such defects results in localized ablation, thus causing a LIPSS discontinuity. In this case, we notice that ablation followed by the redeposition of the ablated material may result in the formation of random structures as high as 500 nm, i.e., ∼2.3 times bigger than the original Mo film thickness.

### 3.2. Effect of Irradiation Spots Overlaps

The 0.3×0.15 mm surface regions were irradiated in a matrix arrangement by varying $O_{x}$ and $O_{y}$ in the ranges 92.3–95.7% and 63.1–75.4%, respectively, using uni-directional scanning at $F_{p}$ = 0.266 J/cm${}^{2}$ (see Table 1).

**Figure 3:**
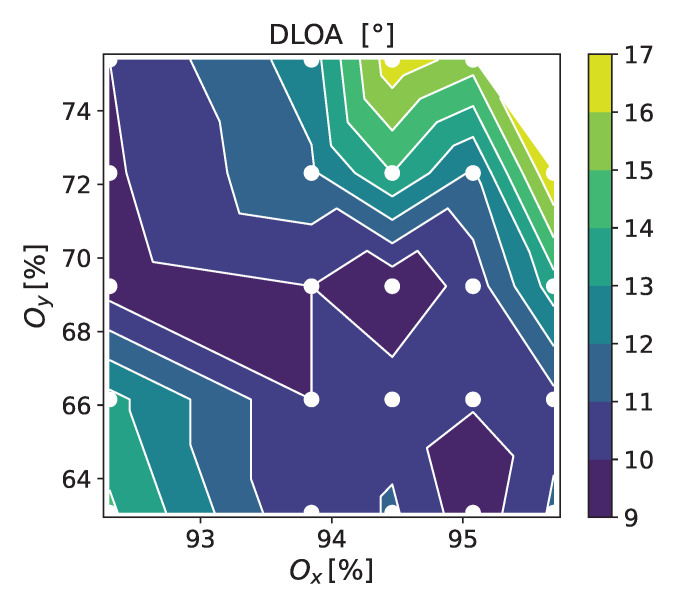
Map of the DLOA values for LIPSS fabricated on the Mo thin films at $F_{p}$ = 0.266 J/cm${}^{2}$ and uni-directional scanning as a function of $O_{x}$ and $O_{y}$. The laser polarization was parallel to the scanning direction. The map is created by interpolation between the DLOA of 24 different irradiations, each marked by a white dot.

For this peak fluence, which enables a satisfactory homogeneity and regularity of the LIPSS, we analyzed the DLOA for different raster scans over the Mo film surface with the polarization parallel to the scanning direction. Figure 3 shows the measured DLOA values. The data were calculated using the ImageJ software [30] with an OrientationJ plugin [31] by analyzing 24 SEM images. This gives the trend in the overlaps $O_{x}$ and $O_{y}$, where minimum DLOA values can be obtained on our sample. For 0.266 J/cm${}^{2}$ within the studied range of overlaps, the high regularity region is roughly located along the line $O_{y}∼3.78-3.3\;O_{x}$. This relation allows a coarse determination of a wider range of pairs ($O_{x}$, $O_{y}$), which can be used for the fabrication of HR-LIPSS at this fluence.

### 3.3. Effect of the Scanning Direction

**Figure 4:**
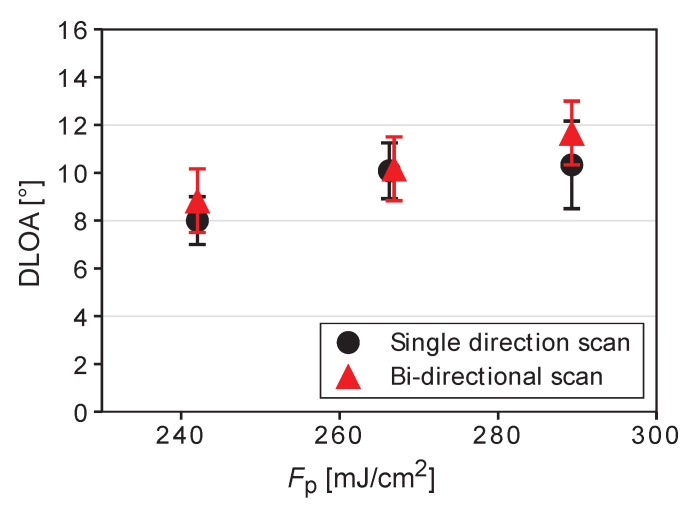
DLOA values for the cases of uni- and bi-directional laser scanning ($O_{x}$ = 95.1% and $O_{y}$ = 66.2%) at three different laser fluences.

A uni-directional laser scanning was used in all previously discussed measurements. However, bi-directional scanning is faster and can be preferable if it provides the LIPSS DLOA values similar to those obtained using the uni-directional scanning technique. To study the effect of the scanning direction on the LIPSS regularity, uni- and bi-directional laser scanning was performed at three laser fluences for the case of polarization parallel to the scanning direction. The results are presented in Figure 4. A small, but noticeable degradation of the LIPSS quality upon changing the scanning to the bi-directional mode is seen for the laser fluences tested. We also note the higher regularity for the peak fluence of 0.242 J/cm${}^{2}$ compared to 0.266 J/cm${}^{2}$. It is indeed expected that this multiparametric investigation for LIPSS inscription can be further refined to achieve better conditions. However, the trends evidenced here should be the same.

### 3.4. Analysis of Chemical Composition and Possibility of Film Dewetting

**Figure 5:**
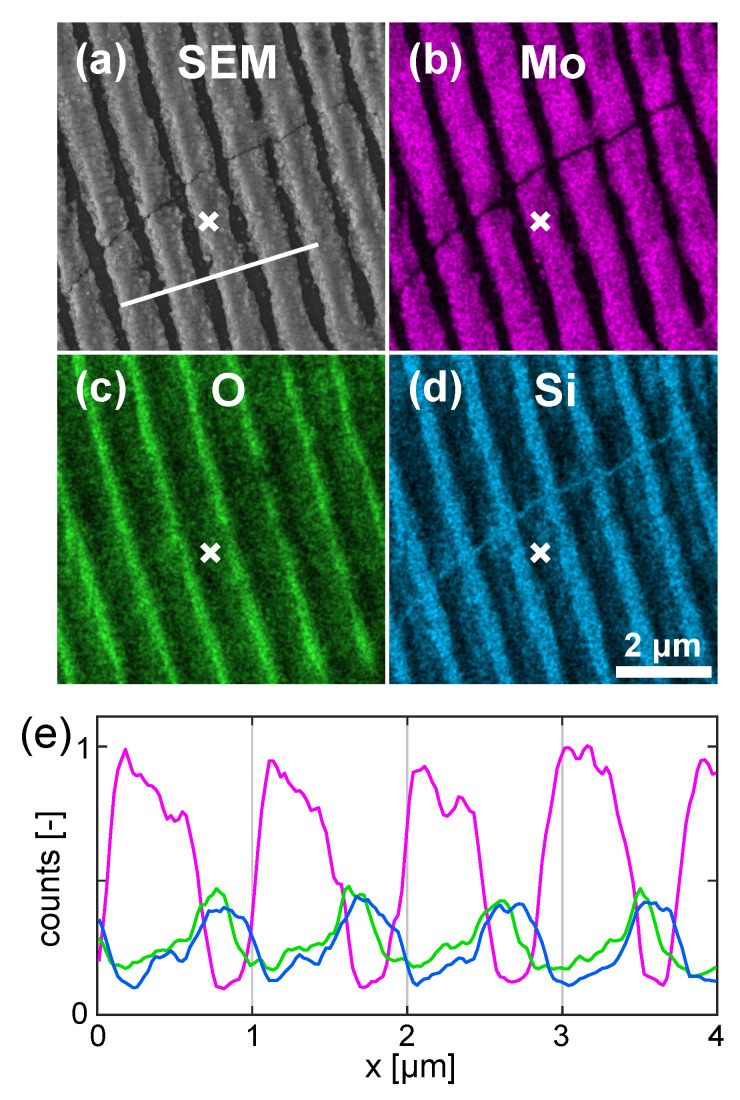
(**a**) Magnified view of the Figure 2a. (**b**–**d**) EDX maps showing Mo, Si, and O contents respectively. (**e**) Chemical composition measured across the white line marked in (**a**). Colors of the lines correspond to the colors in (**b**–**d**). The white crosses are added for guiding an eye, marking the same position in all maps.

**Figure 6:**
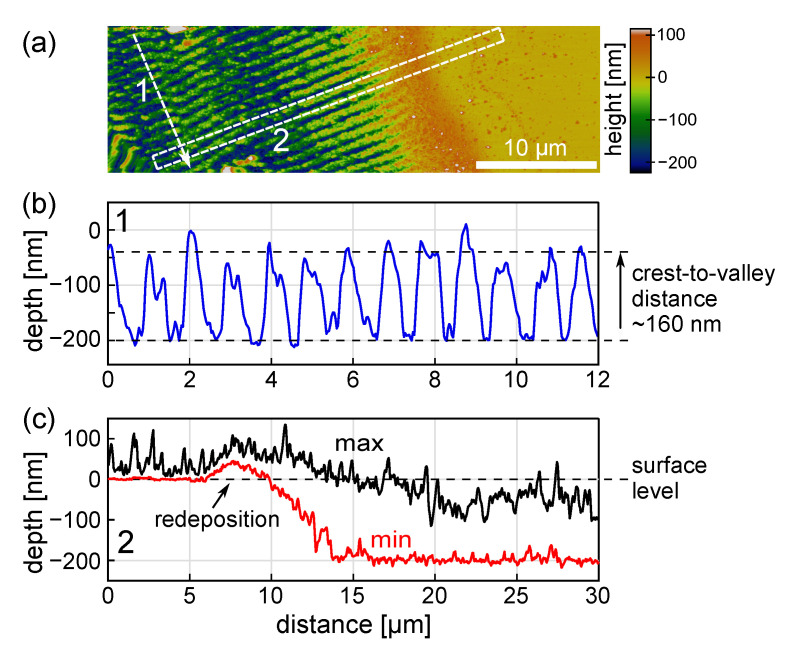
(**a**) AFM image of the LIPSS obtained under the same irradiation conditions as in Figure 2a, showing an edge of the scanned region. (**b**) Surface relief along the line 1 in (**a**). (**c**) The minima (red line) and maxima (black line) of the structured area within the rectangular region 2 in (**a**) along its long side are given to illustrate how the depth of the structures is evolving from the virgin surface towards the regular LIPSS region.

Figure 5a presents a magnified view of the HR-LIPSS image shown in Figure 2a. The EDX analysis (Figure 5b–d) demonstrates that the LIPSS crests consist mostly of Mo, while the substrate components are dominating in the valleys. It should be noted that, upon ablation in air, an oxide layer can be formed on the top of the ablated metal. However, according to Santagata et al. [3], this effect becomes important for molybdenum at fluences above 10 J/cm${}^{2}$, which is much higher than applied in our experiments. Indeed, Raman analysis shows only some traces of MoO${}_{2}$ (not presented here). This is also confirmed by Figure 5e, where the variation of chemical composition is shown along the white line in Figure 5a. Such component profiles may point to an effect of the metal film dewetting observed in a number of studies [32,33,34,35,36]. To gain insight into the possibility of film dewetting, a topographical AFM analysis (see Figure 6) of the edge of a laser scanned area was performed for the same irradiation conditions as in Figure 2a.

The LIPSS profile along the line 1 of the AFM scan in Figure 6b is typical for the main (middle) part of the scanned area. We also demonstrate the evolution of the scanned area depth within region 2 (Figure 6c), which shows the edge effect, where the effective number of pulses per spot is decreasing (note that the LIPSS are perpendicular to the scanning direction). Along this region, the depth of the scanned area is gradually increasing toward its saturation at a distance of ∼10 µm from the non-irradiated zone. This is observed on both the minimal structure depth (red line) and its maximal height (black line). The scanned area is also surrounded by a rim of redeposited materials (Figure 6c).

Within the scanned area, except for its ∼10-µm edges, the crest level of the LIPSS is approximately 40 nm deeper than the pristine film surface (Figure 6c), which points to an ablative regime of LIPSS formation. The crest-to-valley height of the periodic structure is ∼160 nm. Interestingly, for certain pulse overlap conditions (not shown), the valleys of the LIPSS can be slightly deeper than the initial level of the substrate, indicating the damage of fused silica in the valley zones. The damage can be attributed to the ablation of silica being heated by molten molybdenum, whose melting temperature is much higher than that of fused silica (2897 K vs 2005 K respectively), while the recoil pressure of the ablation products forces molten material to move to the sides of the strong ablation zone [37]. Other effects that can influence the substrate deepening in the valley zones upon laser irradiation are the compaction of fused silica [38,39] and possibly a Marangoni convection [40].

Thus, the EDX maps (Figure 5) and the topological analysis (Figure 6) show that the LIPSS valley bottoms are almost clean from molybdenum and, hence, dewetting may play a role in the formation of the LIPSS relief. It should be noted that a more detailed AFM analysis of 5 µm × 5 µm area of the LIPSS (not shown here) revealed that some amount of molybdenum ablated upon the LIPSS formation redeposits to the valleys in a form of nanoparticles. The structured area becomes semitransparent, which can be observed by the naked eyes. Below, a theoretical analysis is presented to gain insight into the laser heating level and the temperature dynamics of the Mo film to appraise a possible material evolution.

## 4. Discussion

### 4.1. Two-Temperature Modeling of a Single-Pulse Action

**Figure 7:**
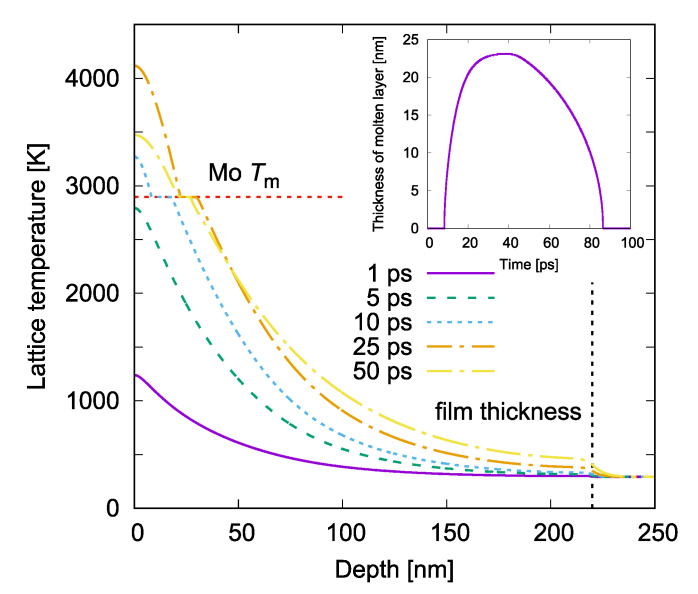
Evolution of the lattice temperature profile toward the depth of the laser-irradiated Mo film for different time moments counted from the laser pulse maximum (color online). The TTM simulations were performed for a single laser pulse action at the irradiation conditions of the present experiments ($F_{p}=0.267$ J/cm). The melting temperature of $T_{m}=2897$ K is marked by the red dotted line. The black dashed vertical line shows the Mo-glass interface. The laser pulse comes from the left. In the inset, the evolution of the molten layer thickness is given.

We performed one-dimensional two-temperature modeling (TTM) for our irradiation regimes (220 nm Mo film on a glass substrate irradiated by a Gaussian laser pulse at λ=1030 nm and τ=1.4 ps). The details of our numerical model are presented elsewhere [39,41]. Calculations were carried out for a single laser pulse action. The calculated evolution of Mo lattice heating is presented in Figure 7 for the laser fluence $F_{p}=0.267$ J/cm${}^{2}$. In this regime, the maximal lattice temperature of 4133 K is reached at 22 ps after the laser pulse maximum, whereas the maximal depth of the completely molten layer of 23 nm is achieved later, at 38 ps.

The temperature level, well above the melting point, may lead to the ablation of the molten layer via the spallation mechanism [42]. The thermodynamic critical point of Mo is >12,000 K [43] and hence the mechanism of phase explosion cannot be realized [44]. Due to the intrinsic intensity modulation of the linearly polarized laser light on the surface roughness resulting in a periodic laser energy absorption along the material surface [45], the melting and ablation of a surface layer may proceed in a periodic manner [46,47,48].

According to the simulations, the maximum melting depth induced by a single pulse action is approximately ten times smaller than the film thickness. However, during the scanning process under the conditions with $F_{p}=0.266$ J/cm${}^{2}$, $O_{x}=92.3\;\%$, and $O_{y}=75.4\;\%$, the effective number of laser pulses per irradiation spot is N≃53. It is thus expected that the LIPSS are deepening by the successive laser pulses [49] due to, for example, a resonant coupling and/or laser light scattering on the periodic surface relief.

In this scenario, in the LIPSS valleys at a certain laser pulse, the film can be molten down to the film–substrate interface. As a result, the nanoscopic molten layer between LIPSS ridges may experience dewetting as mentioned above with the relocation of molten material towards colder/unmolten ridges. We can expect that dewetting can widely be observed upon the periodic nanostructuring of thin films and be useful for a variety of applications (see for example, Ref. [50]). However, the dewetting effect upon LIPSS formation has to depend strongly on the film thickness and irradiation conditions [51]. It should be also noted that, in the ablative regimes, hydrodynamic instabilities contribute to an enhancement of the LIPSS profiles [52,53].

### 4.2. Insights from Surface Plasmon Polaritons Theory

In Ref. [15], it was demonstrated that the DLOA has a strong correlation with the mean free path $L_{\mathrm{SPP}}$ of surface plasmon polaritons (SPP) excited on surface roughness features. In short, when incoming laser light is scattered on the surface roughness, the SPP are generated and propagate along the surface according to light polarization. These surface electromagnetic waves are interfering with incoming light, thus inducing periodic laser energy absorption and hence the modulated heating of a surface layer of material [45]. However, if the $L_{\mathrm{SPP}}$ is large, a considerable interference occurs between the SPP generated on different surface features, causing a randomized distribution of the absorbed laser energy and thus reducing the LIPSS regularity. Hence, the shorter the $L_{\mathrm{SPP}}$ (or lesser interference between SPP), the higher the LIPSS regularity. It is why lossy materials (for which the imaginary part of the dielectric permittivity is relatively high and the $L_{\mathrm{SPP}}$ is small [15,54]) enable much better structuring quality than low-loss ones [15].

Depending on the film thickness, the SPP can be generated either at the interface between air and film [54] (for optically thick films) or additionally at the film-substrate interface. For the latter case, a thin-film SPP model should be used to predict both the $L_{\mathrm{SPP}}$ and the LIPSS periodicity (see [55]). In our case, the 220 nm Mo film is optically thick (the optical penetration depth $\alpha^{-1}=\lambda /\left(4\pi k\right)≃19.5$ nm for a Mo extinction coefficient *k* = 4.2 at λ = 1030 nm [56]), and the theory for bulk materials [54] should be used. We obtain a mean free path of SPPs $L_{\mathrm{SPP}}≃$ 4.3 µm. This small value of $L_{\mathrm{SPP}}$ points out that highly regular LIPSS can be formed on Mo surfaces under our irradiation conditions.

The spatial period of the modulated electromagnetic field $\Lambda_{\mathrm{SPP}}$ [15], which represents an evaluation of the LIPSS periodicity in ideal conditions based on a simplified formula for the SPP period, is estimated as $\Lambda_{\mathrm{SPP}}≃$ 1019 nm. The calculated $\Lambda_{\mathrm{SPP}}$ value overestimates the LIPSS periods shown in Figure 2 by ∼10%. This discrepancy can be related to the number of pulses employed in this work, as increasing the number of pulses induces a pulse-to-pulse shift of the resonant SPP mode at the surface of the formed nanostructure [57,58,59,60,61].

### 4.3. Comment on Other Factors Influencing LIPSS Regularity

We note that the minimum DLOA value obtained under our irradiation conditions is higher than those obtained previously by Gnilitskyi et al. [15] using femtosecond laser pulses, namely 8° (Figure 4) vs. 5.3°. This can be due to several factors. First, the irradiation spot size can influence the LIPSS regularity. In our case, the $1/e^{2}$ spot diameter is threefold larger than in [15]. For small irradiation spots, there is less interference between SPP generated by different surface roughness features. Indeed, there are indications that smaller DLOA values are typically obtained for smaller irradiation spots as summarized in [15]. The second factor can be the laser pulse duration, which is 6.5 times longer in our case as compared to [15]. It can be speculated that, at longer laser pulses, the absorbed laser energy delocalizes already during laser irradiation due to electron heat conductivity, thus leading to a less pronounced temperature modulation [47] that in its turn influences the effects of subsequent pulses. However, the effects of pulse duration on LIPSS regularity have not yet been investigated. Other factors are the stability of the galvo scanner, which shows small but noticeable pulse-to-pulse deviations from a straight line in our case, and quality of the deposited films. We notice that, although the surface roughness of the films is similar in [15], in our case, the film crystallinity can differ for different deposition conditions, thus resulting in some difference in the LIPSS regularity.

## 5. Conclusions

With the example of Mo thin films, we performed a systematic study of highly regular LIPSS formation using the picosecond laser PERLA-B of the HiLASE Centre. The effects of laser spot overlaps upon beam scanning over the film surface, pulse energy, and scanning method were investigated. With a proper choice of the irradiation conditions, highly regular structuring was achieved on lossy metal films. A theoretical analysis of laser-induced film heating was carried out based on two-temperature modeling, which allowed to reveal the film melting dynamics and to evaluate the ablation mechanism. The LIPSS regularity was also discussed based on the surface plasmon polariton theory. Finally, the question on the possible film dewetting was raised. As a whole, this work outlines the procedure that can be used for the fabrication of high-quality periodic surface structures on large areas.

## Figures and Tables

**Table 1:** Overview of the parameters investigated in this work.

Series of Tests	$O_{x}$ [%]	$O_{y}$ [%]	$F_{p}$ [J/cm${}^{2}$]	Angle between Polarization and Scanning Direction [°]	Uni- or Bi-Directional Scanning	Smallest DLOA in the Series [°]
Regime 1, Section 3.1	92.3	75.4	0.266	0, 12, 90	uni-directional	9.1±1.4
Regime 2, Section 3.2	92.3–95.7	63.1–75.4	0.266	0	uni-directional	9.3±1.7
Regime 3, Section 3.3	95.1	66.2	0.242–0.289	0	uni- and bi-directional	8±1

## Data Availability

Not applicable.

## Abbreviations

The following abbreviations are used in this manuscript:

AFM	Atomic Force Microscope
DLOA	Dispersion of the LIPSS Orientation Angle
FFT	Fast Fourier Transform
HR-LIPSS	Highly Regular LIPSS
LIPSS	Laser-induced Periodic Surface Structures
SEM	Scanning Electron Microscope
SPP	Surface Plasmon Polaritons
